# Spatial variation and determinates of dietary diversity among children aged 6–23 months in Ethiopia: spatial and multilevel analysis using Ethiopian Demography Health Survey (EDHS) 2019

**DOI:** 10.1186/s13690-022-00905-3

**Published:** 2022-06-06

**Authors:** Tewodros Getaneh Alemu, Masresha Asmare Techane, Chalachew Adugna Wubneh, Nega Tezera Assimamaw, Getaneh Mulualem Belay, Tadesse Tarik Tamir, Addis Bilal Muhye, Destaye Guadie Kassie, Amare Wondim, Bewuketu Terefe, Bethelihem Tigabu Tarekegn, Mohammed Seid Ali, Beletech Fentie, Almaz Tefera Gonete, Berhan Tekeba, Selam Fisiha Kassa, Bogale Kassahun Desta, Amare Demsie Ayele, Melkamu Tilahun Dessie, Kendalem Asmare Atalell

**Affiliations:** 1 Department of Pediatrics and Child Health Nursing, School of Nursing, College of Medicine and Health Sciences, University of Gondar, Gondar, Ethiopia; 2 Department of Community Health Nursing, School of Nursing, College of Medicine and Health Sciences, University of Gondar, Gondar, Ethiopia

**Keywords:** Dietary diversity, Inadequate minimum dietary diversity, Children, Spatial analysis, Multilevel analysis, Ethiopia

## Abstract

**Background:**

Minimum dietary diversity is the consumption of five or more food groups from the eight World Health Organization recommended food groups. Adequately diversified diet, in terms of amount and composition, is critical for optimal growth, development, and long-term health outcomes in the first 2 years. Understanding the regional variation of dietary diversity and the underlying factors is crucial for developing and implementing interventions. However, the use of spatial approaches in dietary studies has not been widely established. Therefore, this study aimed to explore the spatial patterns and determinates of minimum dietary diversity practice among 6–23 months children in Ethiopia.

**Methods:**

Secondary data analysis was conducted based on the Demographic and Health Surveys data conducted in Ethiopia. A total weighted sample of 1578 children aged 6–23 months was included for this study. The Global Moran’s I was estimated to look into the regional variation of dietary diversity and hotspot and cold spot areas. Further, multivariable multilevel logistic regression was used for factor analyses. Adjusted Odds Ratio with 95% CI was used to declare the strength and significance of the association.

**Results:**

Overall, 87.4% (95% CI: 85.7 to 88.9%) of children in 2019 had inadequate Minimum dietary diversity. We identified statistically significant clusters of high inadequate dietary diversity (hotspots) notably observed in Somali, Afar, Eastern and western Amhara, western Tigray, Benishangul, and Northeastern and western parts of the southern nations, nationality and peoples’ regions. Inadequate dietary diversity was significantly higher among young children, uneducated mother, married women, younger mother, no postnatal check, community with higher level of poverty and community level uneducated woman.

**Conclusion:**

According to the findings of this study inadequate Minimum dietary diversity for children as measured by World Health Organization dietary assessment shows high. Children's dietary diversity was distributed non-randomly in different districts across Ethiopia's regions. The findings of the study provided critical evidence about dietary diversity and associated factors. Hence, policy should focused on improve education status of Mother, boosting economic status of the community, increased maternal continuum of care and focused on young children nutrition may advance dietary diversity.

## Background

Minimum dietary diversity is the consumption of five or more food groups from the eight recommended food groups, namely breast milk, grains, roots, and tubers, legumes, and nuts, dairy products (infant formula, milk, yogurt, cheese), flesh foods (meat, fish, poultry, and liver/organ meats), eggs, vitamin A- rich fruits and vegetables, other fruits and vegetables, for higher dietary quality and to meet daily energy and nutrient requirements [1, 2]. Dietary diversity is one way of conceptualizing dietary adequacy and optimal nutrient intake [3]. Adequately diversified diet, in terms of amount and composition, is critical for optimal growth, development, and long-term health outcomes in the first 2 years [4]. The World Health Organization (WHO) has established guidelines concerning Infant and Young Child Feeding (IYCF) practices for children aged 6–23 months by considering Minimum Dietary Diversity (MDD) as one of the core eight indicators [5].

Globally, only a few children are receiving nutritionally adequate and diversified foods[6]. In many countries, less than one-fourth of infants aged 6–23 months meet the criteria for dietary diversity [2]. Children who do not receive sufficient dietary diversity after 6 months of age are at risk of being stunted, despite they have optimum breastfeeding [7]. Fourteen percent of children have an adequately diverse diet, and only 8% of non-breastfed children in Ethiopia have received a diversified diet [8]. Globally, 149 million children younger than 5 years old were stunted during 2020, with 75% of such children living in the African and Southeast Asia Region [2]. In Ethiopia, the prevalence of stunting in under-five children is 37% [8]. More than two-thirds of malnutrition- related child deaths are associated with inappropriate feeding practices during the first two years of life [9].

An inadequate diversified diet characterized by the deficiency of macronutrients and micronutrients results in poor nutritional status indicated by stunting, underweight and wasting as well as there will be frequent childhood illnesses like diarrheal diseases and infections [10]. Moreover, nutritional deficiencies during this period can lead to impaired cognitive development, growth retardation, smaller adult stature, and a consequence of compromised educational achievement and low economic productivity which become impossible to reverse later in life [11–13].

In low income countries place of residence, age of the child, maternal education, birth order, wealth index and the number of children less than five years old in the household, high burden of disease and mal-feeding practices, home gardening and media exposure were some of the factors which determine minimum dietary diversity [11, 14–16]. Spatial study conducted in the 2011 EDHS indicated that A large area of the Amhara region, some parts of Afar, Somali, a few parts of Tigray, BenishangulGumuz, and Gambella were areas of inadequate dietary diversity [17].

Sustainable Development Goal-2 (SDG-2) agenda, which aims to end malnutrition in all its forms by 2030 [18]. In cognizance of this, the government of Ethiopia has initiated Growth and Transformation Plan II, Second National Nutrition Program (NNP II), and Seqota Declaration. Nonetheless, the prevalence of under nutrition in 6–23 months is still high [8].

There are studies conducted on the determinants of minimum dietary diversity practice among 6–23 months children in Ethiopia. However, the risk areas (hot spot) of minimum dietary diversity practice among 6–23 months children are not identified. Thus, this study aimed to explore the spatial patterns of minimum dietary diversity practice among 6–23 months children in Ethiopia. Therefore, detecting the geographic variation of minimum dietary diversity during this age is important to prioritize and design targeted intervention programs to reduce inadequate minimum dietary diversity practice especially in those areas with a consistently higher risk of inadequate minimum dietary diversity practice over time.

## Methods

### Study design

A secondary analysis of EDHS 2019 was done to investigate the breadth of spatial patterns of food diversity and associated factors among Ethiopian children aged 6–23 months. The Demographic Health Survey (DHS) is a multi-round cross-country survey that evaluates population health with a focus on maternal and child health, as well as population health indicators of global importance. The data was gathered in collaboration with Ethiopia's Central Statistical Agency (CSA) and the Ministry of Health. A stratified two-stage cluster sampling technique was applied. A total of 305 enumeration areas (EAs) were chosen in the first stage, with 105 probabilities proportionate to EA size (93 in urban regions and 212 in rural areas) (based on the 2019 EPHC frame). A fixed number of 30 households in each cluster were chosen in the second stage. Used Kids Record (KR) files, which contain information about women and children, for this specific research, and extracting important variables related to inadequate dietary diversity were performed from the data set. In this study, children aged 6–23 months during the survey period were included. Therefore, the total weighted sample size from data analyzed in this study was 1578.

### Study setting

The study was conducted in Ethiopia, a country located in the horn of Africa geographical coordinates9.145° N latitude and 40.4897° East longitude [19]. The country has an estimated total surface area of 1,126,829km2. As a landlocked country, it is bordered by Djibouti, Eritrea, Kenya, Somalia, South Sudan, Sudan, and Somaliland (Somalia). Agriculture is the predominant economic activity accounting for over 83.9% of its Gross Domestic Product (GDP), these agricultural activities are mainly dependent on rainfall. Each of the country’s nine administrative regions and two administrative cities is organized into zones, districts, towns, and kebeles (the smallest administrative units) [20].

### Study tool and measurement

#### Dependent variables

The dietary diversity indicator utilized in the analysis was constructed using data from the Demographic Health survey’s 24-h recall of food groups [21]. The dietary diversity score for children aged 6–23 months is defined as the proportion of children aged 6–23 months who consumed foods from at least five of the eight food groups within a 24-h period, which are 1-grains, roots and tubers, 2-legumes and nuts, 3-dairy products, 4-meat, fish, poultry, and liver/organ meats, 5-eggs, 6-vitamin A-rich fruits and vegetables, 7-other fruits and vegetables, and 8-breast milk, according to WHO and UNICEF guidelines. In this study, the minimum dietary diversity score of the children was divided into two categories. Those children between 6 and 23 months who consumed at least five food categories in the last 24 h before an interview are considered to have met the minimum dietary diversity requirements, their minimum dietary diversity score categorized as having adequate minimum dietary diversity. Furthermore, children aged between 6 and 23 months who consumed less than five food categories in the last 24 h before an interview were considered to have not met the minimum dietary diversity requirements, their minimum dietary diversity score categorized as having inadequate minimum dietary diversity.

#### Independent variables

The independent variables were identified from various literatures and these cover factors related to child, maternal, household, and community characteristics that impact children’s diet [12, 22–24]. The child characteristics included sex, age in months. The household and maternal characteristics included under five, number of children in household, sex of the household head, mother educational level, wealth index, marital status, age of the women in years, number of families, child lives with whom, and religions. Health-related characteristics including birth order, postnatal check within 2 months, place of delivery, number of Anti Natal Care (ANC) visit, and twin child and community-level variables included region, residence, community-level wealth, and community-level education status. Community level women education and community poverty level were constructed through the aggregation of individual-level factors to conceptualize their neighborhood effect on inadequate dietary diversity. The aggregated community level predictor variables were constructed by aggregating individual level values at cluster level, and binary categorization (high or low) of the aggregated variables were done based on the distribution of the proportion values calculated for each cluster (community). Median for not normally distributed community level aggregated predictor variables were used as cut off point for categorization. Histogram was used to check the distribution whether it is normal or not.

#### Data management and analysis

To restore the survey’s representativeness the sample weights were applied to compensate for the unequal probability of selection between the strata. We used in STATA v.14 to weight the survey data and create descriptive and summary statistics for non-spatial analysis. A spatial map was also produced for visual presentation of dietary diversity at the regional and district level using ArcGIS version 10.8 and Sat Scan version 9.6. The district demarcation shape file for Ethiopia was obtained from the central statistical agency database.

#### Spatial autocorrelation analysis

The global spatial autocorrelation (Global Moran’s I) statistic measure was used to assess whether MDD among children was dispersed, clustered, or randomly distributed in the study area. It was used to detect the spatial autocorrelation of minimum dietary diversity: calculated Moran’s I values close to − 1 indicate inadequate dietary among children are dispersed, whereas I close to + 1 indicate inadequate dietary clustered and inadequate dietary among children is distributed randomly if I value zero. A statistically significant Moran’s I (*p* < 0.05) indicates the presence of spatial autocorrelation.

#### Hot spot analysis

Spatial analysis tools SaTScan and ArcGIS were used to perform final confirmatory spatial studies. The SaTScan identifies areas geographically where significant higher aggregate rates. Its results show hotspot areas in circular windows, showing that distributions inside the windows are higher than predicted when compared to the distributions outside of the cluster windows. Hot spot areas with a high cluster of inadequate dietary diversity and Cold spot areas with low-level clusters were identified in ArcGIS. Hotspot analysis of minimum dietary diversity was done used Geti Ord Gi* statistics for each area, across Ethiopia. The statistical significance of clustering is determined by the Z-score, and the significance is determined by the p-value. If the z-score is between − 1.96 and + 1.96, the p-value would be larger than 0.05, then the null hypothesis cannot be rejected; the pattern displayed is most likely be the result of random spatial processes. If the z-score is outside the range, the observed spatial pattern is likely too unique to be due to chance, and the p-value will be significant. A high Gi* statistical output indicates a “hotspot,” while a low Gi* indicates a “cold-spot.”

#### Spatial interpolation of minimum dietary diversity among children

To anticipate the unsampled from sampled values, the spatial interpolation approach was used. For predicting and producing smooth surfaces of childhood minimum dietary diversity, the kriging interpolation approach was utilized. As a result, in this study, ordinary kriging was employed to evaluate the burden of inadequate dietary diversity among children.

#### Multilevel logistic regression

A two (i.e., the individual and community) levels, a multilevel logistic regression model was fitted to investigate factors associated with minimum dietary diversity. In the analysis, four models were fitted. The null model, which contain only the outcome variable, assesses the degree of the intra-cluster variation of minimum dietary diversity. The first model contains individual-level variables, the second model contains only community-level variables, and the third model contains both individual-level and community-level variables. A *P*-value of < 0.05 was used to define statically significance. Adjusted Odds Ratios (AOR) with their corresponding 95% confidence intervals (CIs) were calculated to identify the independent predictors of minimum dietary diversity. To evaluate the variation between clusters, the intra-cluster correlation coefficient (ICC), Median Odds Ratio (MOR), and proportional change in variance (PCV) statistics were calculated. MOR is a measure of unexplained cluster heterogeneity, while ICC was employed to explain cluster variation. PCV measures the total variation attributed by individual-level factors and community-level factors in the multilevel model as compared to the null model PCV. For Model comparison log-likelihood and deviance were used.

## Results

### Spatial autocorrelation

The spatial autocorrelation analysis showed that, there is a significant spatial variation of inadequate minimum dietary diversity among children age 6–23 months in Ethiopia. In this study the Global Moran’s index value, *p*-value and Z-score values were 0.19, 0.000075 and 3.96 respectively. This indicates that inadequate minimum dietary diversity in Ethiopia had a significant spatial dependence.

### Hotspot analysis of inadequate minimum dietary diversity

**Fig. 1:**
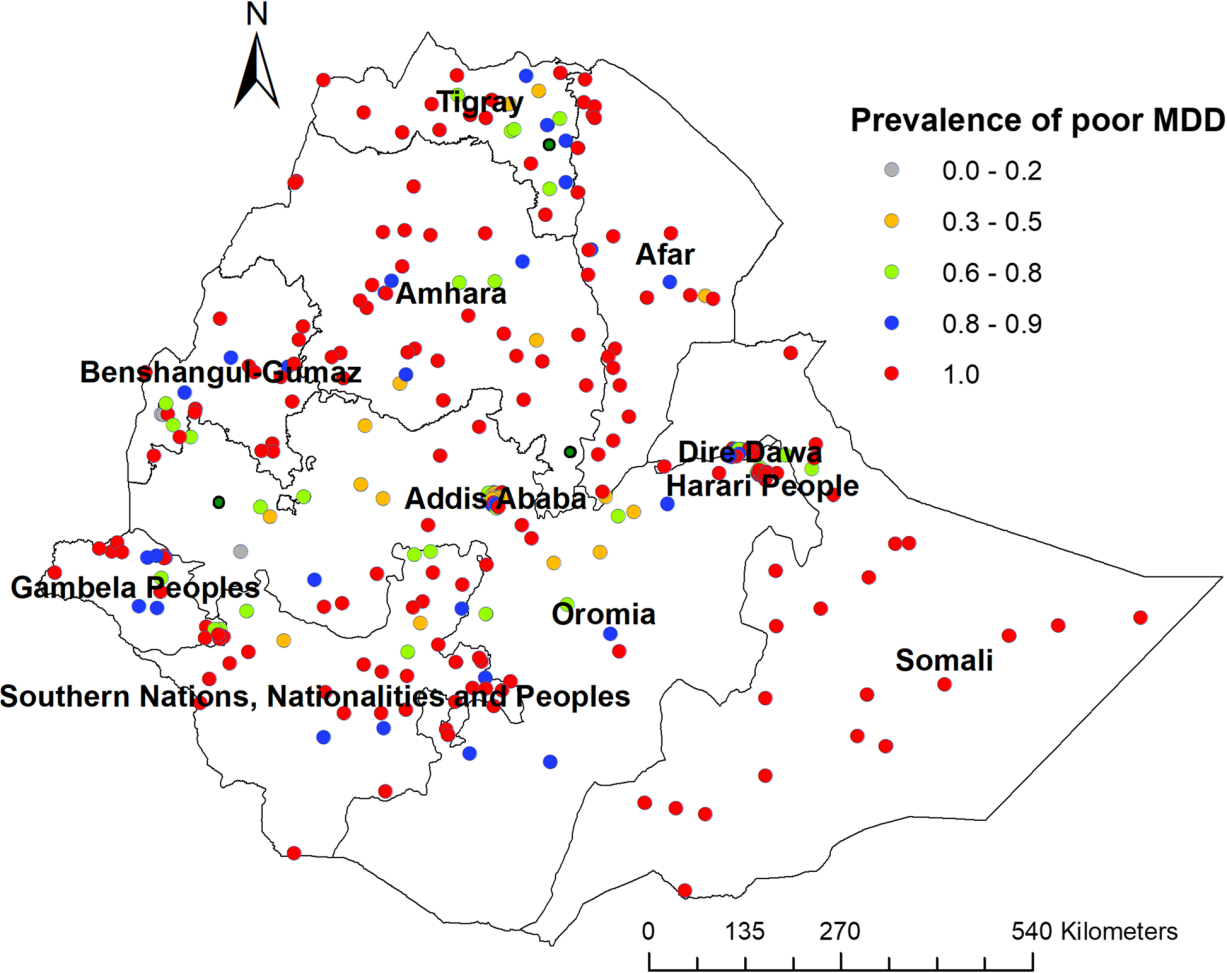
Cluster and outlier analysis map of inadequate dietary diversity among children in Ethiopia, 2019

In this study, significant clustering of inadequate minimum dietary diversity was observed in Somali, Afar, Eastern and western Amhara, western Tigray, Benishangul, and Northeastern and western parts of the southern nations, nationality and peoples’ regions (Fig. 1).

**Fig. 2:**
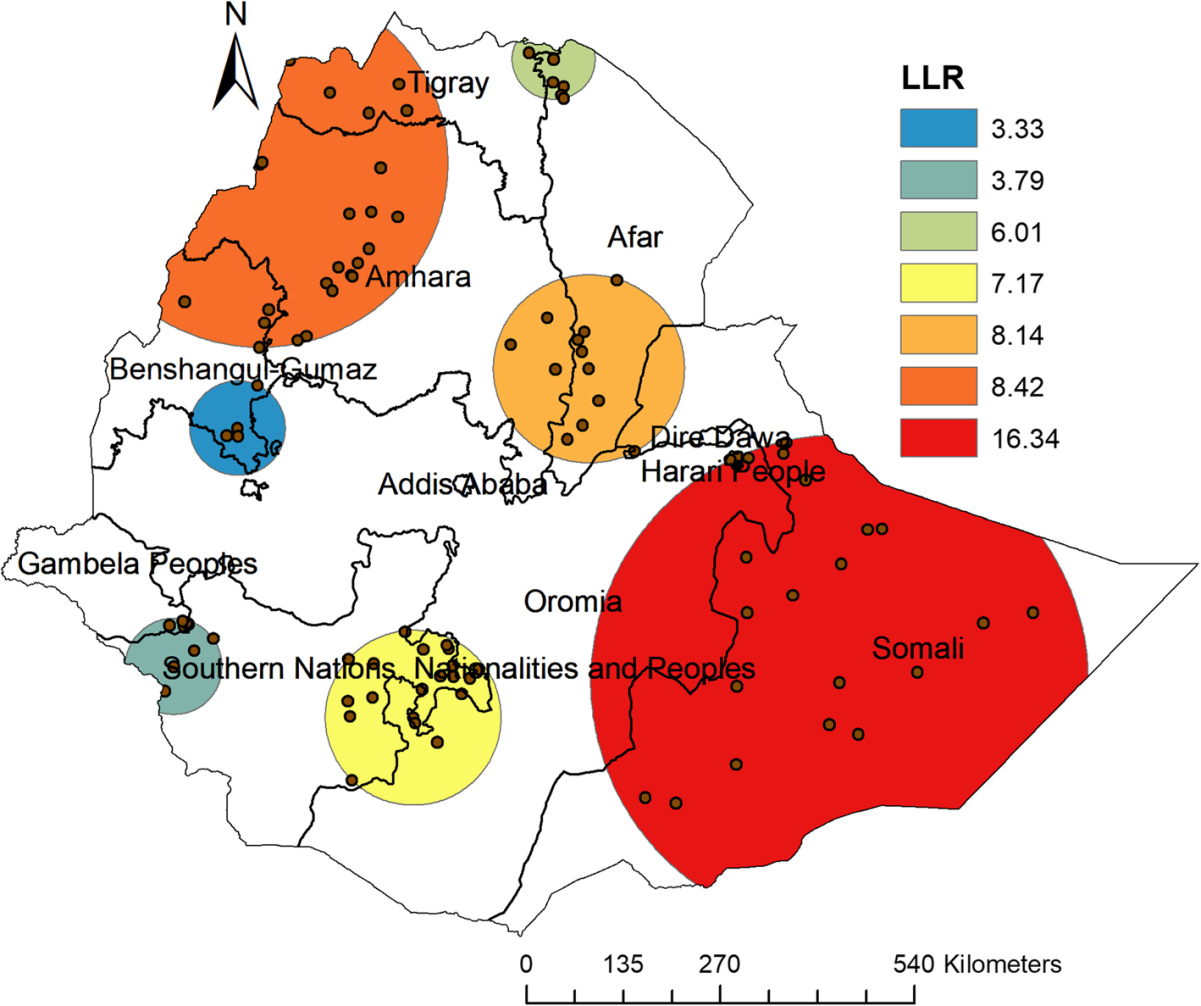
SaTScan analysis map of inadequate dietary diversity among children age 6–23 months in Ethiopia, 2019

In the spatial Sat scan analysis, a total of 50 significant clusters were detected in the sat scan windows, of which 27 were primary (most likely) clusters and 23 secondary clusters (Table 1). The primary sat scan window was detected in Somali, Eastern Oromia and Harari peoples’ regions at 6.505335 North, 43.486778 East, a radius of 343.63 km, a total population of 170 and 169 cases of inadequate minimum dietary diversity, with a RR of 1.13 and LLR 16.34 (*P*-value = 0.000027). This indicates that children in this sat scan window were 1.13 times at experience high risk to have inadequate minimum dietary diversity as compared to children out of the windows. The secondary sat scan window was detected in Western Tigray, Western Amhara and Northwestern Benishangul regions located at 12.985634 North, 36.239465 East, with a radius of 254.43 km, RR of 1.11 and LLR 8.42 (*p*-value = 0.042) (Fig. 2).

**Table 1:** Significant Spatial Clusters of Inadequate Dietary Diversity among Children in Ethiopian Demography Health Survey, 2019 (*n* = 1578)

Cluster	Enumeration areas (cluster detected)	Coordinates(radius)	Population	Cases	R.R	LLR	*P*-value
1	123, 138, 137, 135, 145, 136, 134, 131, 142, 140, 122, 133, 132, 141, 124, 129,125, 121,250,107, 248, 249, 130, 255, 247, 254,244	6.505335 N, 43.486778 E/343.63 km	170	169	1.13	16.34	0.000027
2	85, 55, 21, 84, 4, 56, 22, 82, 74, 75, 57, 59, 54, 81, 83, 53, 9, 159, 8, 162, 79, 80, 163	12.985634 N, 36.239465 E/254.43 km	123	121	1.11	8.42	0.042
3	50, 49, 48, 43, 68, 47, 40, 64,42, 66, 33, 127,	10.425370 N, 40.353666 E/130.74 km	70	70	1.13	8.14	0.052
4	172, 188, 115, 89, 197, 182, 113, 186, 199, 181, 190, 198, 183, 184, 185, 187, 117, 178, 191, 202	6.066850 N, 38.154184 E/ 121.51 km	129	126	1.10	7.17	0.120
5	39, 35, 15, 37, 27,38	14.300432 N, 39.911829 E/ 55.93 km	52	52	1.12	6.01	0.328
6	215, 200, 216, 228, 224, 223, 222, 227, 201	6.708096 N, 35.156792 E/66.83 km	33	33	1.12	3.79	0.954
7	167, 168, 169, 164	9.684778 N, 35.956673 E/65.52 km	29	29	1.12	3.33	0.995

### Spatial interpolation of inadequate dietary diversity

**Fig. 3:**
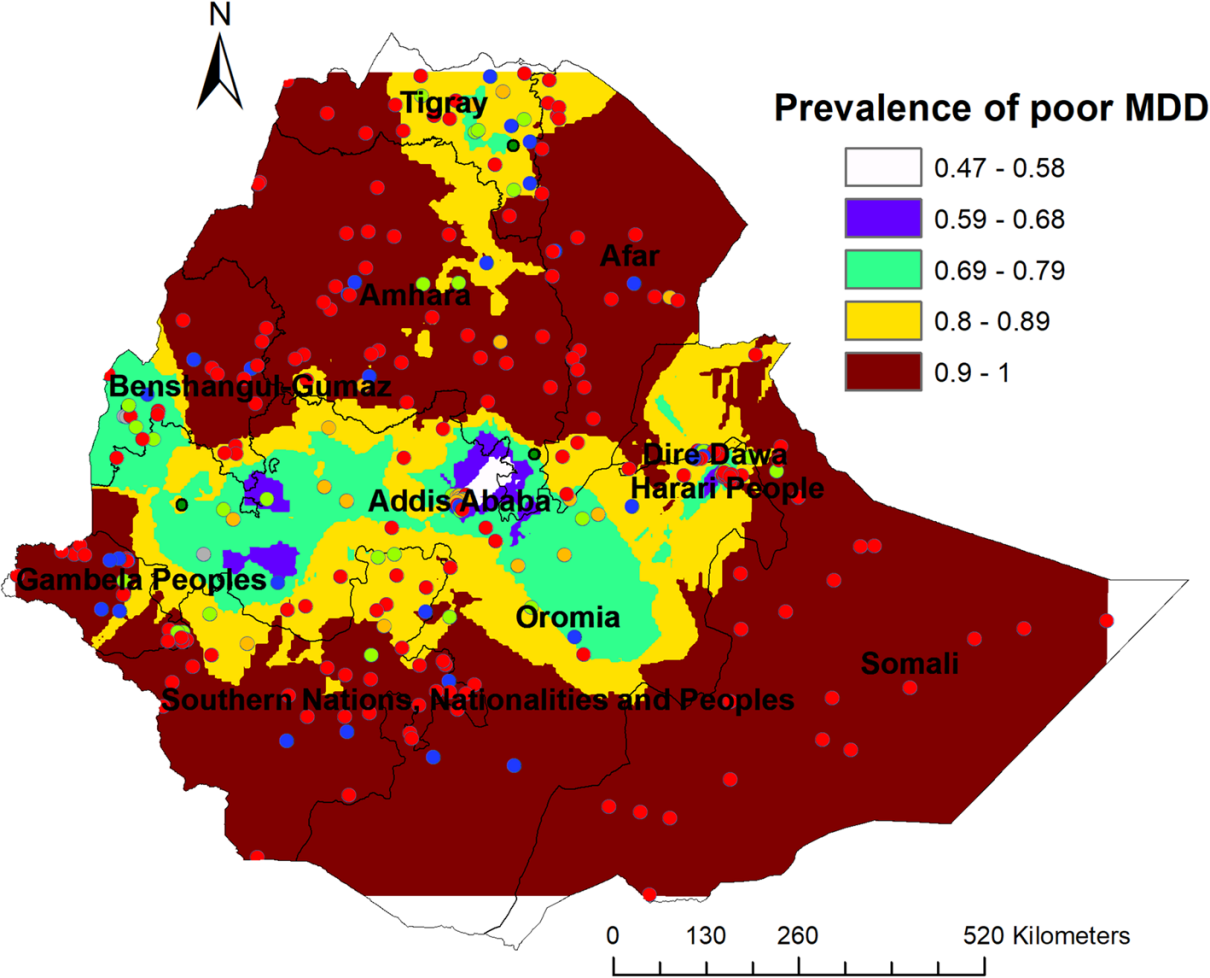
Kriging interpolation map of inadequate dietary diversity among children age 6–23 months in Ethiopia, 2019

The ordinary kriging interpolation map was used to estimate the inadequate minimum dietary diversity in Ethiopia in an unsampled area. High inadequate minimum dietary diversity was observed in Western Tigray, Amhara, Afar, Northern Benishangul, Gambela, Somali and Southern Nations, nationalities, peoples’ regions. Whereas, low predicted inadequate minimum dietary diversity prevalence was observed in Oromia, Tigray and western Benishangul regions (Fig. 3).

### Socio-demographic and economic characteristics of participants

From the DHS KR dataset, a total of 1578 children aged 6– 23 months were included in this study. Half (52.1%) of the children were males. Near to seventy percent of the children were between the age of 6–11 and 12–17 months. The median age of the children was 14 (IQR 10, 18) months. The majority of mothers were currently married 1490(85.5%). Approximately 41% of the mothers had primary education. Around 41.3% of the children were born from poor household wealth status (Table 2).

**Table 2:** Socio-demographic and economic characteristics of children age 6–23 month and their caregiver in Ethiopia Demography Health Survey, 2019 (*n* = 1578)

Variables	Categories	Weighted Frequency	Percentage
Age of the child	6-11 m	507.61	32.17
	12-17 m	570.91	36.18
	18-23 m	499.50	31.65
Child sex	Male	821.39	52.05
	Female	756.63	47.95
Number of under-5 children in the household	2 or less	1418.84	89.91
	3 and above	159.18	10.09
Sex of the household head	Male	1358.79	86.11
	Female	219.22	13.89
Mother education level	No education	711.54	45.09
	Primary	646.86	40.99
	Secondary	134.37	8.52
	Higher	85.24	5.40
Wealth index	Poor	651.72	41.30
	Middle	298.76	18.93
	Rich	627.53	39.77
Marital status	No married	87.20	5.53
	Married	1490.81	94.47
Age of women in year	15–24	506.38	32.09
	25–34	788.67	49.89
	35–49	282.96	17.93
Number of household members	< = 5	872.88	55.32
	6 and above	705.13	44.68
Child lives with whom	Mother	1490.91	98.97
	Lives else where	15.58	1.03
Religion	Orthodox	574.39	36.40
	Catholic	6.55	0.42
	Protestant	433.56	27.48
	Muslim	534.59	33.88
	Tradition	25.86	1.06
	Other	8	0.50

### Health-related characteristics of mothers and child characteristics

Of the total women, 44.8% of women had four and above ANC follow-up. More than half (54.4%) of mothers were delivered at health facilities. Nearly half (46.5%) of children were 2^nd^to 4^th^birth order (Table 3).

**Table 3:** Health-related characteristics of mothers and child in Ethiopia Demography Health Survey, 2019 (*n* = 1578)

Variables	Categories	Weighted Frequency	Percentage
Birth order	First order	391.49	24.81
	Second to fourth order	734.30	46.53
	Fifth and above order	452.22	28.66
Postnatal check within two months	No	1320.54	86.60
	Yes	204.39	13.40
Place of delivery	Institution delivery	858.24	54.39
	Home delivery	719.77	45.61
Number of ANC visit	No visit	379.74	24.90
	1–3 visit	462.85	30.34
	4 and above visit	682.72	44.76
Child is twin	No	1543.13	97.79
	Yes	34.89	2.21

### Community-level variables in Ethiopia DHS

Near to three fourth of children (72%) were rural dwellers. More than half (53.6%) were in the lower community-level poverty. The highest number 1366 (86.6%) of children were from the large central region (Table 4).

**Table 4:** Community-level variables in Ethiopia Demography Health Survey, 2019 (*n* = 1578)

Variables	Categories	Weighted Frequency	Percentage
Community level poverty	Higher	731.62	46.36
	Lower	846.39	53.64
Community level education	Higher	549.69	34.83
	Lower	1028.32	65.17
Region	Metropolitan	65.17	4.13
	Small peripheral	145.92	9.25
	Large central	1366.92	86.62
Residence	Urban	441.14	27.96
	Rural	1136.88	72.04

### Prevalence of minimum dietary diversity

The dietary diversity determined based on a 24 h recall method showed that 87.4% (95% CI: 85.7 to 88.9%) of the children had inadequate MDD (i.e., not receiving the recommended minimum dietary diversity or not feeding five or more food items within 24 h preceding data collection).

### Random effect analysis and model comparison

The ICC value in the null model indicates 45.6% of the total inadequate minimum dietary diversity was due to the difference between clusters. Besides, the high MOR value in the null model which was 4.8 revealed that when we randomly select children from two clusters, children’s from a high-risk cluster had 4.8 times more likely to have inadequate minimum dietary diversity as compared to children from a low-risk clusters. Moreover, the PCV in the final model revealed that about 27% of the variability in inadequate minimum dietary diversity was explained both by individual and community level factors. Regarding model fitness, model IV was the best-fit model since it had the lowest deviance (Table 5).

**Table 5:** Random effect and model fitness for the assessment of inadequate Minimum Dietary Diversity among children in Ethiopia, 2019 (*n* = 1578)

Parameter	Null model	Model 1	Model 2	Model 3
ICC	45.56	45.38	44.93	40.07
MOR	4.84(3.43,7.49)	4.81(3.44,7.38)	4.74(3.35,7.41)	4.09(2.99,6.09)
PCV	Reference	18.8%	20.12%	27%
Model comparison				
Log likelihood	-525.97	-466.26	-505.06	-457.12
Deviance	105,194.69	93,251.69	101,012.72	91.424.71

*ICC* Intra-Cluster Correlation, *MOR* Median Odds Ratio, *PCV* Proportional Change in Variance

### The mixed-effects analysis result

The model with smaller deviance and the largest likelihood (Model IV) was the best fit model for our data and the interpretation of the fixed effects was based on this model. Model IV was adjusted for both individual and community-level factors. Age of child, educational status of the mother, marital status, and postnatal check within 2 months, community-level wealth, and community-level educational status were significantly associated with inadequate minimum dietary diversity among children age 6–23-months in Ethiopia. Inadequate minimum dietary diversity among children aged 12–17 and 18–23 months were 73% (AOR = 0.3, 95% CI: 0.2–0.5), and 57% (AOR = 0.4, 95%CI: 0.3–0.7) less risk as compared with a child aged 6–11 months. Children from mothers with the educational level of primary, secondary and higher education had 57% (AOR = 0.4, 95% CI: 0.3–0.7), 57.7% (AOR = 0.4, 95% CI: 0.2- 0.9) 70% (AOR = 0.3, 95%CI: 0.1- 0.8) less likely to have inadequate minimum dietary diversity as compared with children from non-educated mothers. The odds of inadequate minimum dietary diversity among children from married mothers was 6 times (AOR = 6.2, 95% CI: 2.7–14.1) higher than those of children from not married mothers. Children from mothers aged from 25–34 and 35–49 years old had 44% (AOR = 0.7, 95% CI: 0.3–0.97), 70% (AOR = 0.3, 95% CI: 0.1- 0.7) less likely to have inadequate minimum dietary diversity as compared with children from mother age 15–24 years old. Children from women with a postnatal check within 2 months had 63% (AOR = 0.4, 95% CI: 0.2–0.6) less risk of inadequate minimum dietary diversity compared with children from women with no postnatal check within 2 months. Children from a community with a high level of poorness had 2.7(AOR = 2.7, 95%CI: 1.1–6.8) times higher inadequate minimum dietary diversity than children from a community with a low level of poorness. Children from a community with a high level of un-education had 2.8(AOR = 2.8, 95%CI: 1.1–7.1) times higher inadequate minimum dietary diversity than children from a community with a low level of un-education (Table 6).

**Table 6:** Individual and community level Factors affecting Minimum Dietary Diversity in Ethiopian Demography Health Survey, 2019 (*n* = 1578)

Variables	Categories	MDDs		Odds ratio			
		Adequate	Inadequate	COR	AOR1(95% CI)	AOR2(95% CI)	AOR3 (95% CI)
Age of child	6-11 m	41(8.11)	467(91.89)	1	1		1
	12-17 m	92(16.05)	479(83.95)	0.34(0.21, 0.54)	0.26(0.16, 0.44)***		0.27(0.16, 0.45)***
	18-23 m	65(13.09)	434(86.91)	0.52(0.32, 0.85)	0.42(0.25, 0.71)***		0.43(0.25, 0.73)***
Sex of the household head	Male	170(12.87)	1189(87.49)	1	1		1
	Female	28(12.56)	191(87.13)	0.55(0.32, 0.95)	1.17(0.59, 2.29)		1.08(0.54, 2.15)
Mother education level	No education	54(7.64)	657(92.36)	1	1		1
	Primary	97(15.06)	549(84.94)	0.55(0.36, 0.85)	0.36(0.22, 0.60)***		0.43(0.26, 0.74)***
	Secondary	28(20.65)	107(79.35)	0.45(0.24, 0.83)	0.38(0.17, 0.85)***		0.43(0.19, 0.95)**
	Higher	19(21.82)	67(78.18)	0.25(0.11, 0.54)	0.25(0.09, 0.66)***		0.31(0.12, 0.82)**
Wealth index	Poor	50.67(7.78)	601.05(92.22)	2.42(1.46, 4.01)	1.95(1.05, 3.63)*		1.24(0.62, 2.46)
	Middle	42.30(14.16)	456.46(85.84)	1.76(1.03, 3.01)	1.62(0.87, 3.00)		1.39(0.74, 2.66)
	Rich	105.18(16.76)	522.35(83.24)	1	1		1
Marital status	Not married	27.33(31.34)	59.87(68.66)	1	1		1
	Married	170.82(11.46)	1319.98(88.54)	5.22(2.74, 9.96)	7.28(3.26, 16.28)***		6.19(2.73, 14.07)***
Age of women	15–24	63.63(12.57)	442.74(87.43)	1	1		1
	25–34	96.84(12.28)	691.83(87.72)	0.89(0.59, 1.34)	0.50(0.29, 0.87)***		0.56(0.32, 0.98)**
	35–49	37.68(13.32)	245(86.68)	0.71(0.42, 1.19)	0.25(0.11, 0.56)***		0.30(0.13, 0.69)***
Birth order	First-order	55.85(14.26)	335.67(85.74)	1	1		1
	Second to fourth order	101.79(13.86)	632.51(86.14)	1.10(0.72, 1.69)	1.36(0.78, 2.39)		1.27(0.77, 2.23)
	Fifth and above order	40.54(8.96)	411.68(91.04)	1.61(0.96, 2.69)	2.31(1.01, 5.27)*		1.99(0.86, 4.60)
Postnatal check within two months	No	149.25(11.30)	1171.29(88.70)	1	1		1
	Yes	47.62(23.30)	156.78(76.70)	0.41(0.25, 0.65)	.38(0.22, 0.65)***		0.37(0.22, 0.64)***
Place of delivery	Institution delivery	134.67(15.69)	723.55(84.31)	1	1		1
	Home delivery	63.47(8.82)	656.31(91.18)	1.41(0.89, 2.21)	1.15(0.65, 2.03)		1.05(0.59, 1.88)
Number of ANC visit	No visit	40.83(10.75)	409.19(88.41)	1.05(0.62, 1.77)	.55(0.29, 1.04)		0.53(0.27,1.01)
	1–3 visit	53.65(11.59)	409.19(88.41)	1.15(0.74, 1.78)	1.04(0.63, 1.69)		0.92(0.56, 1.52)
	4 and above visit	102.38(15.00)	580.34(85.00)	1	1		1
Community level poverty	Higher	55.18(7.54)	676.44(92.46)	5.35(2.58, 11.1)		2.58(1.19, 5.58)**	2.73(1.09, 6.80)**
	Lower	142.97(16.89)	703.42(83.11)	1		1	1
Community level education	Higher	30.65(5.58)	519.05(94.42)	6.83(3.12, 14.93)		3.88(1.69, 8.95)***	2.78(1.08, 7.14)**
	Lower	167.50(16.29)	860.81(83.71)	1		1	1
Region	metropolitan	15.71(24.1)	49.46(75.90)	1		1	1
	Small peripheral	4.65(3.19)	141.93(96.81)	14.49(3.51, 59.83)		4.07(0.91, 18.13)	3.41(0.67, 17.34)
	Large central	177.80(13.01)	1189.12(86.99)	3.19(1.26, 8.09)		1.55(0.53, 4.50)	1.41(0.43, 4.59)
Residence	Urban	67.01(15.19)	374.12(84.81)	1		1	1
	Rural	131.14(11.54)	1379.86(88.46)	3.12(1.48, 6.59)		1.13(0.48, 2.71)	0.77(0.29, 2.07)

*ANC* Anti Natal Care, *MDD* Minimum Dietary Diversity

*P value < 0.05, **P value < 0.01, *** P value < 0.001

## Discussion

Minimum dietary diversity is widely regarded as a key indicator of IYCF around the world, and addressing childhood malnutrition by using this complimentary feeding approach is highly recommended. Improvements in IYCF practices are crucial to the promotion of childhood nutrition.

In this study, the proportion of inadequate minimum dietary diversity was 87.44% (95%CI: 85.7 to 88.9%) of children aged 6–23 months in recent 2019 EDHS. This is an alarming sign that indicates the problem is still a huge nutritional concern that requires due attention to be paid by designing comprehensive nutritional strategies.

This finding was consistent with studies conducted in Somalia and other studies conducted in Ethiopia [24–26]. However, it’s higher than studies conducted Nepal, Indonesia, and Tanzania [12, 27, 28]. The possible discrepancy is due to low food production and consumption, as well as people's purchasing capacity in Ethiopia for a variety of food goods. The recent increase in the price of consumable products, as well as a lack of understanding about how to prepare complementary foods, may contribute to Ethiopian children's inadequate dietary diversity. On the other hand, the finding is a bit lower than a study done in rural Burkina Faso [29]. The possible discrepancy might be the study participants in Burkina Faso were from a rural area where as this study was done national wide and include rural and urban residences.

The spatial distribution of inadequate MDD significantly varies across Ethiopia with a Global Moran’s Index values of 0.19 at *p*-value < 0.001 [22, 30]. Consistent to previous studies conducted in Ethiopia significant clustering of inadequate minimum dietary diversity was observed in Somali, Afar, Amhara, western Tigray, Benshangul-Gumuz, and the southern nations, nations and peoples’ regions [22]. This highlighted that the problem is still present, and that neither the government nor other stakeholders had made any effective interventions. High inadequate minimum dietary diversity was observed in Somali, Afar, Amhara, Western Tigray, Benshangul-Gumuz and the southern nations, nations and peoples’ regions both in hotspot analysis and cluster outlier analysis. Household food insecurity was high in these region of the country [31]. The eastern (Somali and Afar) part of the Ethiopia is frequently affected by drought, which implicate the high inadequate MDD in these regions [32]. Furthermore, educational, health care and water access were low in this parts of the country that will improve the minimum dietary diversity level of children [33].

The random-effects logistic regression model indicated that the variation in the inadequate minimum dietary diversity of children’s age 6 to 23 months was attributed to both individual- and community-level factors. The proportional change in variance for the final full model (model IV) revealed that both individual- and community-level factors accounted for about 27% of the variation observed for inadequate minimum dietary diversity of children’s age 6 to 23 months. Similar findings were also found in study done in EDHS 2011 [23].

Educational status of the mother was associated with inadequate minimum dietary diversity of children’s age 6 to 23 months. Mothers who had primary, secondary and higher education level were 57%, 57.7% and 70% more likely feed recommended diversified food to their children respectively than those who had no formal education. This finding is similar with that of prior studies conducted in Ethiopia and Indonesia [28, 34, 35]. It indicates that a higher educational level allows mothers to have better nutrition knowledge for all family members, especially for the children [36]. Another argument is that educated mothers have better employment and higher income, giving more opportunities to purchase diverse foods [13]. Therefore, the government should make policies to cover uneducated mothers, such as in the long term, improvements in education leading to higher levels of mother education can result in better dietary diversity practices. In the short term, programs to improve those practices need to target families with low levels of maternal education and design promotional materials that take account low mother levels of education.

The age of the child was associated with inadequate minimum dietary diversity of children’s age 6 to 23 months. A child age 12–17 and 18–23 months were 73% and 57% more likely received diversified food respectively than a child aged 6–11 months. This study also supported by studies conducted in Ethiopia, Indonesia, Seri Lank and Nepal [24, 25, 27, 28, 37]. This result indicates the relationship between different food groups by age group which implies that food group’s increase as the child age increase. This may be due to mothers’ perceptions toward IYCF [27]. As a result, National strategies and programs targeting mothers with younger children to promote optimal food and feeding practices are necessary.

Mothers who had postnatal check with in two months were 63% more likely feed recommended diversified food than mother who had no postnatal check with in two months. This finding is concurrent with other studies conducted in Ethiopia [25, 38, 39]. It might be do you to mothers who have attended PNC visits may be more informed, have greater access to services and may be from a well off family, and thus more likely to be able to afford and provide of foods more diversified to their children[40].

In the community level characteristics, wealth status was significant predictor of inadequate minimum dietary diversity of children’s age 6 to 23 months. Children’s from higher level of poor community were 2.7 times more likely to feed inadequate minimum dietary diversity than children from lower level of poor community. There have not been any further studies explaining this issue: it may be related to improvement of wealth has a significant effect on food security of the community and play a crucial role and could affect individual and community level nutritional access and status; economic status of the community closely related to community income and financial constraints can be limiting factors for the community to have adequate nutritious food to their children [28, 41, 42]. Therefore the government should work on healthy economy that improves a population’s ability to produce, distribute and consume a sustainable supply of nutritious food.

Children’s from higher level of uneducated community were near to 3 times more likely to feed inadequate minimum dietary diversity than children from lower level of uneducated community. There have not been any further studies explaining this issue: it may be related to education is vital part of a comprehensive health education program and empowers community with knowledge and skills to make healthy food [43]. Education status of the community is closely related to understanding and capable of improving different issues; uneducated community is limited understand and capable to make healthy food and diversified choices[44]. Therefore the government should work on education that improves a population’s understand and ability and making opportunity.

### Strength and limitation

It may not also accurately reflect children past feeding experience since it considers only 24-h feed and we utilized dietary diversity as a dichotomized variable since it is convenient programmatically; however, utilizing dietary diversity as a continuous variable would be fascinating to observe the link and it would be good to understand which components of the diet are underrepresented. Moreover, this study might be affected from small sample size effect because the DHS data for this age specification with an issue is limited. However, the majority of the confidence intervals are quite narrow. In spite of these limitations, the strength of our study lies in the unique characteristics of the DHS. The DHS are nationally representative, and allows for findings to be generalized across the entire country.

## Conclusion

According to the findings of this study inadequate minimum dietary diversity for children as measured by WHO dietary assessment shows high. The spatial distribution of inadequate minimum dietary diversity in this survey was not random. Large area of the Amhara region, some part of Tigray, Somalia region, a few parts of southern nations, nationality and peoples’ region and Benishangul regions were continued to be areas of inadequate dietary diversity. The study found that both the individual and community level factors were associated with the outcome variable. Young children, uneducated mother, married women, younger mother, no postnatal check, community with higher level of poverty and uneducated were significantly associated with minimum dietary diversity among children aged 6–23 months in Ethiopia. The fact that the spatial distribution and statistical association were both well supported by one another established the distribution's scientifically rigorous. The socio-demographic factors observed, as well as districts with poor feeding practices, can be used to build localized intervention and prevention programs to enhance children's feeding practices. Furthermore, it’s also critical in properly allocating resources to increase young children's consumption of a variety of foods. Congruently, the community's educational status and postnatal check of the mother should be improved, and the community's economic position must be improved. Finally, in addition to ongoing efforts to fight child under nutrition, Ethiopia’s Federal Ministry of Health (FMOH) should focus on policies and strategies that favor young children; this means the government should develop new policies and strategies to improve the nutrition of young children (ages 6 to 11 months).

## Data Availability

Data for this study were sourced from Demographic and Health surveys (DHS) and available at: https://www.dhsprogram.com/Data using the detail in the methods section of the document.

## Abbreviations

ANC: Anti Natal Care
AOR: Adjusted Odds Ratio
CI: Confidence Intervals
COR: Crude Odds Ratio
CSA: Central Statistical Agency
DD: Dietary Diversity
DHS: Demographic and Health Survey
EDHS: Ethiopian Demographic and Health Survey
EA: Enumeration Area
EPHC: Ethiopian Public Health Institute
FMOH: Federal Ministry of Health
GDP: Gross Domestic Product
IYCF: Infant and Young Child Feeding
ICC: Intra-Cluster Correlation
ICCM: Integrated Community Case Management
KR: Kids Record
LLR: Log Likelihood Ratio
MOR: Median Odds Ratio
MDD: Minimum Dietary Diversity
NNP: National Nutrition Program
PCV: Proportional Change in Variance
PNC: Post Natal Care
SDGs: Sustainable Development Goals
RR: Relative risk
UNICEF: United Nation International Children Emergency Fund
WHO: World Health Organization
